# Ultrathin 2D Inorganic Ancient Pigment Decorated 3D‐Printing Scaffold Enables Photonic Hyperthermia of Osteosarcoma in NIR‐II Biowindow and Concurrently Augments Bone Regeneration

**DOI:** 10.1002/advs.202101739

**Published:** 2021-08-02

**Authors:** Chao He, Caihong Dong, Luodan Yu, Yu Chen, Yongqiang Hao

**Affiliations:** ^1^ Shanghai Key Laboratory of Orthopedic Implants Department of Orthopedic Surgery Clinical and Translational Research Center for 3D Printing Technology Shanghai Ninth People's Hospital Shanghai Jiao Tong University School of Medicine Shanghai 200011 China; ^2^ Department of Ultrasound Zhongshan Hospital Fudan University Shanghai 200032 China; ^3^ Materdicine Lab School of Life Sciences Shanghai University Shanghai 200444 China

**Keywords:** 3D‐printing, Egyptian blue, osteogenesis, osteosarcoma, photonic hyperthermia

## Abstract

Osteosarcoma (OS) is the primary malignant bone tumor. Despite therapeutic strategies including surgery, chemotherapy, and radiotherapy have been introduced into the war of fighting OS, the 5‐year survival rate for patients still remains unchangeable for decades. Besides, the critical bone defects after surgery, drug‐resistance and side effects also attenuate the therapeutic effects and predict poor prognosis. Recently, photothermal therapy (PTT) has attracted extensive attention featuring minimal invasiveness and high spatial‐temporal precision characteristics. Herein, an ultrathin 2D inorganic ancient pigment Egyptian blue decorated 3D‐printing scaffold (CaPCu) with profound PTT efficacy at the second near‐infrared (NIR‐II) biowindow against OS and enhanced osteogenesis performance is successfully constructed. Importantly, this work uncovers the underlying biological mechanisms that genes associated with cell death, proliferation, and bone development are regulated by CaPCu‐scaffold‐based therapy. This work not only elucidates the fascinating clinical translation prospects of CaPCu‐scaffold‐based PTT against OS in NIR‐II biowindow, but also demonstrates the potential mechanisms and offers a novel strategy to develop the next‐generation, multifunctional tissue‐engineering biomaterials.

## Graphical abstract

Herein, the authors develope an ultrathin 2D ancient pigment Egyptian blue decorated 3D‐printing scaffold with profound PTT efficacy at the second biological window of light against OS and enhanced osteogenesis performance. This work also unveils the underlying biological mechanisms through RNA‐seq that genes related to cell death, proliferation, and bone development are regulated by CaPCu‐scaffold‐based therapy.

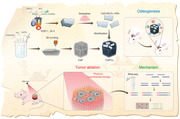

## 1 Introduction

Osteosarcoma (OS) is the primary malignant bone tumor, which mainly develops in children/adolescents (0–19 years old) and older adults (> 65 years old) suffered from Paget's disease of bone or radiation.^[^ ^1^ ^]^ Despite therapeutic strategies including surgery, chemotherapy, and radiotherapy have been introduced into the treatment regimen of OS, the 5‐year survival rate for patients still remains unchangeable for decades.^[^ ^2^ ^]^ Besides, the critical bone defects after surgery, drug‐resistance and side effects also attenuate the therapeutic effects and induce poor prognosis.^[^ ^3^, ^4^ ^]^ Notably, except for the elimination of tumor tissues, reconstruction of the critical bone defects afterward was also unsatisfactory currently and challenging.^[^ ^3^, ^5^, ^6^ ^]^ Hence, more efficient and advanced platforms are instantly needed to be developed for achieving both effective OS elimination and enhanced bone regeneration.

Recently, photothermal therapy (PTT) has attracted extensive attention featuring high spatial‐temporal precision and minimal invasiveness characteristics.^[^ ^7^, ^8^, ^9^, ^10^, ^11^, ^12^, ^13^, ^14^, ^15^ ^]^ PTT regularly depends on photothermal agents (PTAs) to produce sufficient heat irradiated by near‐infrared (NIR) light, leading to a quick localized temperature rise to 42–45 °C for a certain period to ablate cancer cells.^[^ ^16^ ^]^ Up to now, a diversity of PTAs has been introduced into the ablation of tumors, such as organic nanoparticles,^[^ ^17^ ^]^ noble metal nanostructures,^[^ ^18^, ^19^ ^]^ carbon‐based nanomaterials,^[^ ^20^ ^]^ and transition metal chalcogenides.^[^ ^21^ ^]^ Especially, Cu‐containing chalcogenides have featured preferable popularity among numerous PTAs attributing to their intense NIR absorption capability.^[^ ^22^, ^23^ ^]^ Besides, it is noteworthy that Cu^2+^ can not only inhibit cancer cells,^[^ ^24^, ^25^ ^]^ but also enhance osteogenesis,^[^ ^26^, ^27^, ^28^ ^]^ which is more valuable to be introduced into the treatment of OS compared with the commonly studied PTAs that only possess photothermal conversion abilities.

Fortunately, Egyptian blue (EB, CaCuSi_4_O_10_), the oldest known synthetic pigment used in various ancient artifacts (e.g., paintings, wall paintings, tombs, mummies, coffins, etc.),^[^ ^29^ ^]^ is a Ca, Cu, and Si elements‐containing compound which shows the synergistic effects on prompted osteogenesis.^[^ ^30^ ^]^ Besides, EB is also inexpensive and non‐toxic.^[^ ^31^ ^]^ Therefore, EB may have a high potential to facilitate the elimination of OS and the following osseous tissue reconstruction, especially synergizing with the 3D‐printing bioactive scaffolds. After all, it has been widely acknowledged that biomaterials with well‐designed 3D architectures achieved by 3D‐printing may further benefit bone regeneration through temporospatially regulating biochemical and biophysical processes.^[^ ^32^ ^]^

**Figure 1:**
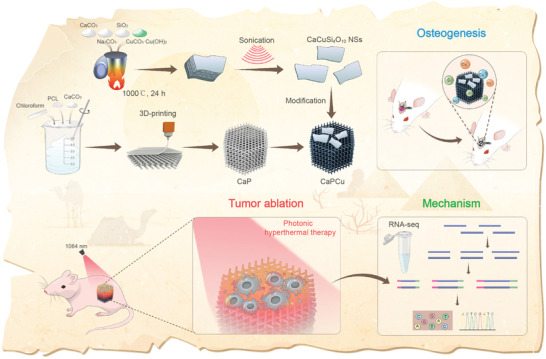
Schematic illustration of the fabrication procedure of CaPCu 3D‐printing scaffolds and the corresponding profound tumor ablation efficacy realized by photonic hyperthermia in NIR‐II biowindow and promoted osteogenesis. Moreover, mRNA sequencing (RNA‐seq) was further utilized to explore the underlying biological mechanisms. It unveiled that both cancer metabolism‐ and bone development‐associated gene terms were enriched after the CaPCu‐scaffold‐based PTT treatment.

Herein, a tactful and multifunctional platform was developed by decorating 2D inorganic EB nanosheets (NSs) on the surface of 3D‐printing CaCO_3_‐PCL scaffolds to achieve both highly effective OS elimination by photothermal ablation under NIR laser irradiation (1064 nm) and the subsequently enhanced bone tissue regeneration (**Figure** **1**). First, EB NSs were obtained by a facile high‐intensity sonication technique. Compared with the bulk materials, the novel 2D NSs exhibit the preferable characteristics in extensive fields (e.g., cancer treatment, multimodal imaging, drug/gene delivery, biosensors, electronics, catalysis, solar cells, optoelectronics, etc.) owing to their unique sheet‐like nanostructures, large specific surface area, specially physicochemical properties and versatile functionalities.^[^ ^33^, ^34^, ^35^ ^]^ As expected, EB NSs display excellent absorption in the second NIR (NIR‐II) biowindow of light and high photothermal‐conversion capability. Compared with the first NIR (NIR‐I) laser (650–950 nm), the NIR‐II laser (1000–1350 nm) possesses lower tissue self‐heating, higher maximum permissible exposure and prompted penetration depth, which is more valuable for clinical application.^[^ ^36^ ^]^ Second, CaCO_3_‐PCL scaffolds (denoted as CaP) were constructed through the personalized 3D‐printing technique based on the well‐defined architecture. In addition, CaCO_3_ and PCL are both FDA‐approved materials to facilitate osseous tissue engineering.^[^ ^37^ ^]^ Third, the EB NSs were then “painted” on the CaP scaffolds (denoted as CaPCu) to integrate the preferable PTT effects and prompted osteogenesis characteristics together. Notably, mRNA sequencing (RNA‐seq) was further utilized to explore the underlying biological mechanisms. It unveiled that both cancer metabolism‐ and bone development‐associated gene terms were enriched after the CaPCu‐scaffold‐based PTT treatment. Therefore, the advanced CaPCu scaffolds exhibit promising clinical translation prospects in the elimination of OS by PTT and subsequent prompted osteogenesis evidenced both in vitro and in vivo.

## 2 Results and Discussion

### 2.1 Structure and Composition Characteristics of CaPCu Scaffolds

**Figure 2:**
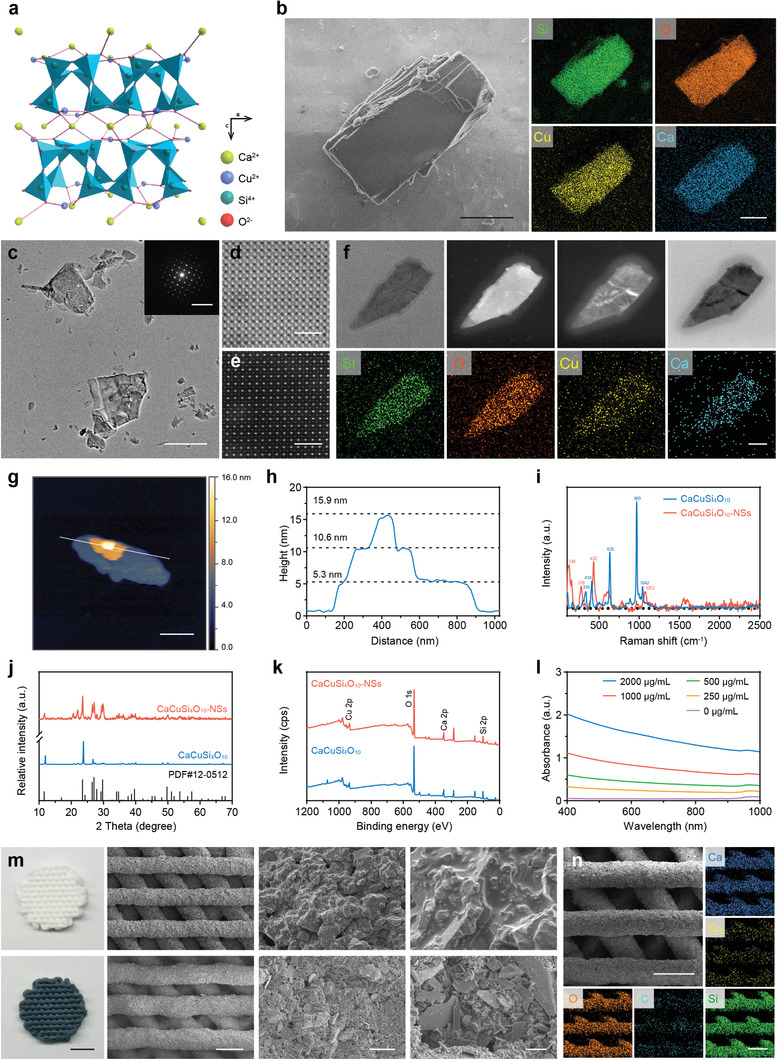
Structure and composition characteristics of CaPCu scaffolds. a) The crystal structure of CaCuSi_4_O_10_. b) SEM pictures and related element composition of multilayer CaCuSi_4_O_10_. Scale bar, 2 µm. c) TEM photographs of ultrathin CaCuSi_4_O_10_ NSs. The inset picture shows the SAED pattern of ultrathin CaCuSi_4_O_10_ NSs. Scale bar, 1 µm. The insert image scale bar, 10 nm^−1^. d) Bright‐field and e) dark‐field STEM pictures of ultrathin CaCuSi_4_O_10_ NSs. Scale bar, 2 nm. f) TEM, dark‐field, and bright‐field STEM photographs of ultrathin CaCuSi_4_O_10_ NSs and the associated element distributions of Si, O, Cu, and Ca. Scale bar, 50 nm. g, h) AFM analysis of CaCuSi_4_O_10_ NSs. Scale bar, 300 nm. i) Raman, j) XRD, and k) XPS spectra of CaCuSi_4_O_10_ and CaCuSi_4_O_10_ NSs. l) vis‐NIR absorbance of ultrathin CaCuSi_4_O_10_ NSs. m) Digital photographs and SEM images of CaP scaffold (upper row) and CaPCu scaffold (lower row). Scale bar from left to right, 3 mm, 500 µm, 25 µm, and 5 µm. n) Surface morphology and relevant element mappings of a CaPCu scaffold. Scale bar, 500 µm.

In order to construct the bioactive CaPCu scaffolds, EB NSs and 3D‐printing CaP scaffolds were obtained at first, respectively. The EB (CaCuSi_4_O_10_) pigment was synthesized by a solid‐state reaction.^[^ ^38^ ^]^ Notably, EB has a special stacked lamellar structure which suggests the potentials of exfoliation to obtain the ultrathin NSs (**Figure** **2**a). Scanning electron microscopy (SEM) photographs and the related elemental analysis further verified the platelet‐like peculiarity with distinct layer structure and the homogeneous distribution of Ca, Cu, Si, and O in bulk EB (Figure 2b). Then, a facile high‐intensity sonication technique was utilized to exfoliate EB into NSs. Transmission electron microscopy (TEM) and high‐resolution TEM images displayed the favorable crystallized ultrathin sheet‐like morphology of EB NSs with square symmetry structure and the corresponding selected‐area electron diffraction (SAED) pattern also demonstrates the well‐defined square symmetry with interplanar angles of 90° (Figure 2c–e and Figure S1a,b, Supporting Information). TEM and the relative elements mapping further confirmed the successful exfoliation of EB into NSs without change of element composition (Figure 2f). Moreover, the thickness and lateral size of single‐layered EB NSs were ≈5.3 and 800 nm analyzed by atomic force microscopy (AFM) and dynamic light scattering (DLS), respectively (Figure 2g,h and Figure S2, Supporting Information). The differences between EB bulk and EB NSs were further determined by Raman spectroscopy, and the downshifted intense peak and the shifty shape confirmed the formation of NSs after exfoliation (Figure 2i). Although X‐ray diffraction (XRD) patterns showed that the EB bulk was successfully synthesized, the intense peaks of EB NSs reduced attributing to the exfoliation (Figure 2j). However, X‐ray photoelectron spectroscopy (XPS) addressed that the exfoliation process did not change the chemical composition, which was in accordance with the former elements mapping analysis (Figure 2k and Figure S3a–d, Supporting Information). Especially, the vis‐NIR spectra of EB NSs show a profound absorption in NIR region, which is beneficial to the magnified PTT (Figure 2l).

The CaP scaffolds were constructed by 3D‐printing. The disordered topography and well‐controlled macropores of CaP and CaPCu scaffolds were observed by digital and SEM images (Figure 2m). Moreover, the macrostructure also endowed the CaPCu scaffolds with preferable compressive strength as 10.65 ± 0.20 MPa (Figure S4, Supporting Information), which was comparable with that of human cancellous bone (2–12 MPa).^[^ ^39^ ^]^ The related element distributions on a CaPCu scaffold further signified the uniform spreading of Ca, Cu, Si, O, and C on its surface (Figure 2n).

### 2.2 Photonic Hyperthermal Characteristics and In Vitro OS Elimination Properties of CaPCu Scaffolds

**Figure 3:**
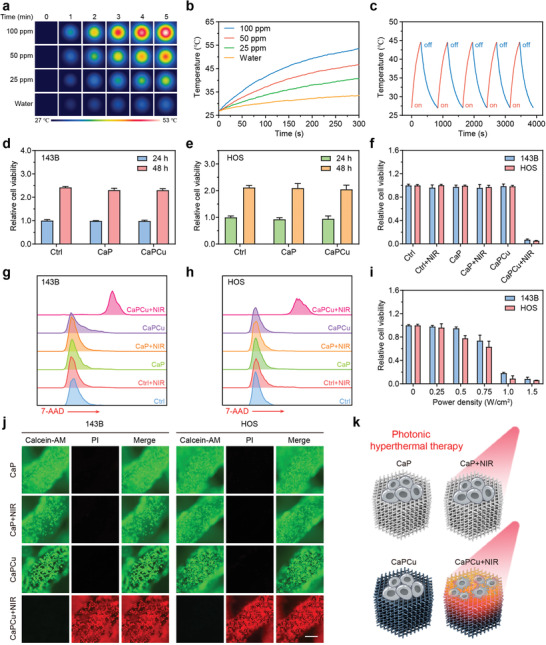
Photonic hyperthermal characteristics and in vitro OS elimination properties of CaPCu scaffolds. a) Time‐dependent photothermal images and b) curves of CaPCu scaffolds soaked at different CaCuSi_4_O_10_ NSs concentrations. c) Photothermal curves of the CaPCu scaffold under 1064 nm laser irradiation for five irradiation on/off cycles. Relative viabilities of d) 143B and e) HOS cells after the indicated treatments for 24 and 48 h (*n* = 3). f) Relative viabilities of OS cells after the indicated treatments (*n* = 3). g, h) Death of OS cells post the indicated treatments for 24 h conducted by FCM. i) Relative viabilities of OS cells for five‐minute irradiation at varied laser power densities (*n* = 3). j) Confocal fluorescence imaging of OS cells after the indicated treatments. Scale bar, 200 µm. k) Scheme indicating CaPCu‐scaffold‐based PTT to eliminate OS.

Since EB NSs featured an intense absorption in NIR region, the in vitro photothermal property of CaPCu scaffolds was further investigated comprehensively under 1064 nm laser irradiation. A loading capacity‐ and power density‐dependent temperature rise profiles were monitored upon exposure to 1064 nm laser (**Figure** **3**a,b and Figure S5a,b, Supporting Information), revealing excellent photothermal effects of CaPCu scaffolds. In detail, the temperature of CaPCu scaffolds was gradually elevated to 48 °C within 5 min at the power density of 1.0 W·cm^−2^. Additionally, the final stable temperature induced by CaPCu scaffolds could be adjusted ranging from 33 to 61 °C under 1064 nm laser irradiation when the power density varied from 0.2 to 1.5 W·cm^−2^ for 5 min. Furthermore, the photothermal capability of CaPCu scaffolds exhibited negligible variation even after undergoing five irradiation cycles, implying favorable photostability (Figure 3c).

Excited by the prominent photothermal conversion performance of CaPCu scaffolds, their PTT effects were further evaluated in vitro. Initially, the biocompatibility of CaPCu scaffolds was assessed. Results indicated that both CaP and CaPCu scaffolds exhibited favorable biocompatibility as measured by the cell counting kit‐8 (CCK8) assay (Figure 3d,e and Figure S6, Supporting Information). Subsequently, the photothermal ablation efficacy of CaPCu scaffolds against OS cells under 1064 nm laser irradiation was analyzed by CCK8 assay and flow cytometry (FCM) (Figure 3f–h). Results elucidated that more than 92% of OS cells were ablated by CaPCu‐scaffold‐based PTT under 1064 nm laser irradiation for 5 min. Moreover, the ablation efficacy showed a power density‐dependent manner (Figure 3i). In order to observe the cell state on the scaffolds after different treatments intuitively, the live and dead cells were stained by calcein‐AM and propidium iodide (PI), respectively (Figure 3j). It can be visualized that OS cells were almost all dead on the CaPCu scaffolds after 1064 nm laser irradiation, while in the absence of laser irradiation, they maintained preferable viability. Meanwhile, OS cells were also survived on the CaP scaffolds independent of laser irradiation. Collectively, these results suggested that both CaP and CaPCu scaffolds were friendly with high biocompatibility and CaPCu scaffolds could serve as desirable PTAs for high‐effective PTT (Figure 3k).

### 2.3 OS Elimination Induced by CaPCu‐Scaffold‐Based PTT In Vivo

**Figure 4:**
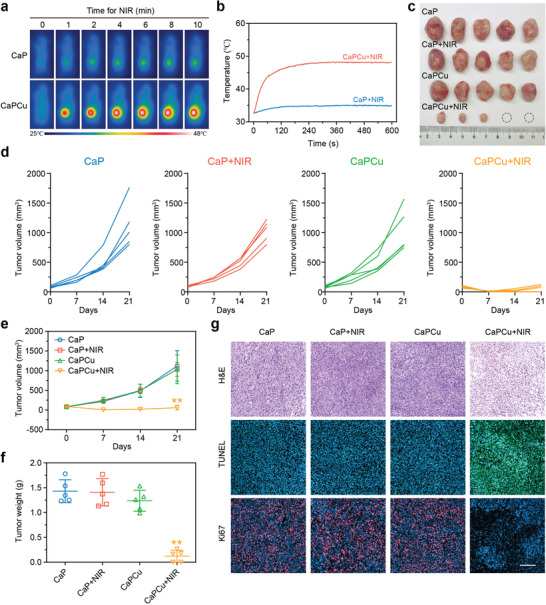
OS elimination induced by CaPCu‐scaffold‐based PTT in vivo. a) Infrared thermal photographs and b) tumor temperature change curves of mice with varied treatments. c) Macroscopic image, d,e) tumor volume and f) weight in various groups (*n* = 5). g) H&E, TUNEL, and Ki67 staining pictures of typical tumors from each group. Scale bar, 200 µm. **P < 0.01 relative to the CaP group.

Encouraged by the astonishing photothermal performance of CaPCu scaffolds in vitro, we then investigated their tumor‐therapeutic potential in vivo. Prior to the evaluation of tumor eradiation efficacy of CaPCu‐scaffold‐based PTT, we first analyzed their biocompatibility in vivo. Fifteen Kunming mice (6–8 weeks) were randomly divided into three groups as Ctrl, CaP, and CaPCu groups with the implantation of indicated scaffolds subcutaneously. After feeding for one month, no significant toxicity was observed among all groups based on blood biochemical and pathological analysis, signifying the favorable biocompatibility of both CaP and CaPCu scaffolds in vivo (Figure S7a,b, Supporting Information). Then, twenty 143B tumor‐bearing nude mice were randomly separated into four groups, including CaP, CaP + NIR, CaPCu, CaPCu + NIR groups. After the implantation of CaP or CaPCu scaffolds for 3 h, 1064 nm laser irradiation for 10 min was applied to the groups involved in the treatment of NIR. Compared with the negligible temperature elevation in CaP + NIR group, the temperature of tumors in CaPCu + NIR group raised rapidly from 33 to 48 °C within 4 min and maintained for another 6 min (**Figure** **4**a,b). The tumor volumes of mice were recorded every week. After the mice were sacrificed, the tumors were resected and photographed (Figure 4c). Combined with the statistical analyses of tumor volumes and weights, it demonstrated that the tumors in the CaPCu + NIR group were almost eliminated by efficient photonic hyperthermia. In stark contrast, tumors in the other three groups were negligibly inhibited (Figure 4d–f).

Moreover, during the experiment period, no unnatural behavior or bodyweight fluctuations were observed, further suggesting the favorable biosafety of various treatments (Figure S8, Supporting Information). Subsequently, the tumors were subjected to pathological evaluation (Figure 4g). Hematoxylin and eosin (H&E) and terminal deoxynucleotidyl transferase dUTP nick‐end labeling (TUNEL) staining both highlighted that there were more cell debris and cell death in the tumors from CaPCu + NIR treated group, accounting for the significant tumor inhibition phenotype in vivo. Meanwhile, tumor cell proliferation was almost suppressed entirely after CaPCu‐scaffold‐based PTT evidenced by the extreme downregulation of Ki67 expression. Collectively, these results assumed that CaPCu‐scaffold‐based PTT could effectively suppress OS progression in vivo without obvious biotoxicity.

### 2.4 Mechanism of CaPCu‐Scaffold‐Based PTT Unveiled by mRNA Transcriptome

**Figure 5:**
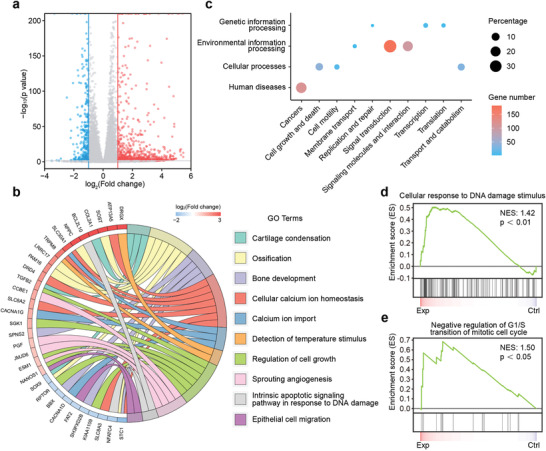
Mechanism of CaPCu‐scaffold‐based PTT unveiled by mRNA transcriptome. a) Volcano map displaying the genes regulated by CaPCu‐scaffold‐based PTT (*P* < 0.05, |fold change| ≥ 2). b) Chord plot, c) KEGG analysis and d,e) GSEA of differentially expressed genes between the Exp and Ctrl groups.

To further probe the underlying mechanism of CaPCu‐scaffold‐based PTT, we performed mRNA sequencing (RNA‐seq). More than 1300 genes were extensively regulated responding to the CaPCu‐scaffold‐based PTT, including 1003 up‐regulated genes and 343 down‐regulated genes (*P* < 0.05, |fold change| ≥ 2) (**Figure** **5**a and Figure S9, Supporting Information). To identify the enriched gene functional terms and signaling pathways involved, Gene Ontology (GO) and Kyoto Encyclopedia of Genes and Genomes (KEGG) analyses were carried out. GO enrichment analysis elucidated that CaPCu‐scaffold‐based PTT not only affected biological processes related to detection of temperature stimulus, regulation of cell growth, sprouting angiogenesis, intrinsic apoptotic signaling pathway in response to DNA damage and epithelial cell migration, but also impacted cartilage condensation, ossification, bone development, cellular calcium ion homeostasis and calcium ion import, inferring the superb PTT efficacy and the potential enhanced osteogenic characteristic of CaPCu scaffolds (Figure 5b and Figure S10, Supporting Information). In addition, pathway analysis based on the KEGG database denoted that CaPCu‐scaffold‐based PTT perturbed several signaling transduction channels such as cell growth and death, cell motility, replication and repair, translation, etc. (Figure 5c). Furthermore, gene set enrichment analysis (GSEA) also elucidated that gene expression profiles associated with cellular response to DNA damage stimulus and regulation of mitotic cell cycle were altered owing to CaPCu‐scaffold‐based PTT (Figure 5d,e). Taken together, these results demonstrated that CaPCu + NIR treatment could not only regulate gene expression patterns correlated with cell death and proliferation in agreement with the pleasurable therapeutic effects observed in vitro and in vivo, but also affect bone development and differentiation.

### 2.5 CaPCu Scaffolds Accelerated Osteogenesis In Vitro and In Vivo

**Figure 6:**
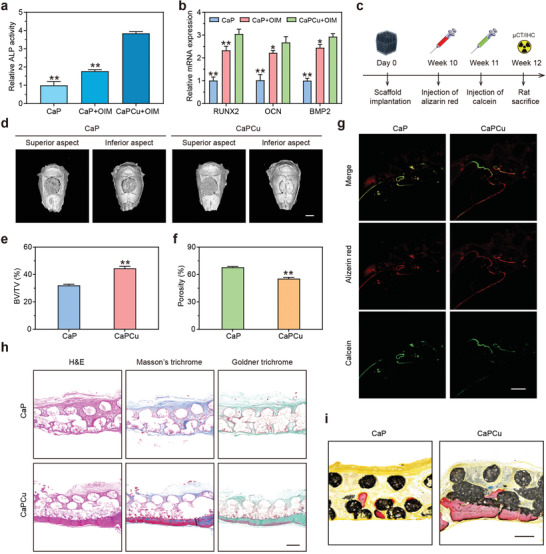
CaPCu scaffolds accelerated osteogenesis in vitro and in vivo. a) Relative ALP activities and b) osteogenic gene expression (RUNX2, OCN, and BMP2) of mBMSCs with the indicated treatments (*n* = 3). *P < 0.05, **P < 0.01 relative to the CaPCu + OIM group. c) Scheme describing the experimental process of skull defect model performed on SD rats (*n* = 5). d) 3D reconstruction of the skull according to data collected by µCT. Scale bar, 5 mm. e) BV/TV and f) the proportion of porosity in CaP and CaPCu groups, elucidating the superior osteogenic functionality of CaPCu scaffolds. **P < 0.01 relative to the CaP group. g) Confocal fluorescence photographs, h) H&E, Masson's, and Goldner trichrome staining, and i) Van Gieson's picrofuchsin staining of skull deficiency area in each group. Scale bar, 500 µm.

Inspired by the clues from RNA‐seq and considering the bioactive effects of Ca, Cu, Si elements, and PCL, the osteogenic peculiarities of CaPCu scaffolds were further assessed in vitro and in vivo. The alkaline phosphatase (ALP) activity on day 7 implied the distinguished osteogenic differentiation of mouse bone mesenchymal stem cells (mBMSCs) on CaPCu scaffolds cultured in the osteoinductive medium (OIM) (denoted as CaPCu + OIM) (**Figure** **6**a). Furthermore, the expression of osteogenic genes (RUNX2, OCN, and BMP2) also up‐regulated, illustrating the desirable osteogenic property of CaPCu scaffolds (Figure 6b).

Moreover, the skull defect rat model was exploited to confer the osteogenic peculiarity of CaPCu scaffolds in vivo (Figure 6c). As designed, CaP or CaPCu scaffolds were implanted and stabilized into the established cranial defects. After 12‐week implantation, more newly generated osseous tissue was observed in the CaPCu group relative to that in the CaP group determined by micro‐computed tomography (µCT) (Figure 6d). Meanwhile, the results of bone volume/total volume (BV/TV) and the percentage of porosity also indicated the distinguishing osteogenic functionality of CaPCu scaffolds (Figure 6e,f). Moreover, confocal laser scanning microscopy (CLSM) analysis stained by alizarin red and calcein was further carried out to evaluate the bone regeneration and mineralization in the groups of CaP and CaPCu scaffolds (Figure 6g). Data demonstrated that the significantly enhanced new bone formation was achieved in the CaPCu group than that in the CaP group. Additionally, the representative H&E, Masson's and Goldner trichrome staining photographs further displayed that much more newborn and mineralized osseous components were acquired in the CaPCu scaffold group relative to that in the CaP group, which is in accordance with the µCT and CLSM analyses (Figure 6h). Likewise, a distinct new bone formation was also realized in the CaPCu scaffold group in comparison with that in the CaP group carried out by Van Gieson's picrofuchsin staining (Figure 6i). It is rational to deduce that the EB NSs were of crucial significance to promote the regeneration of bone in vitro and in vivo. To conclude, it was validated that CaPCu scaffolds featured remarkable osteogenic functionality to accelerate bone regeneration, especially osseous mineralization, which is highly advantageous for the repair of bone defects after the surgery of OS.

## 3 Conclusions

Since the 5‐year survival rate among OS patients has plateaued for decades and far from optimistic,^[^ ^40^, ^41^ ^]^ the development of more effective and novel therapeutic modalities for OS treatment is pretty essential to improve prognosis. In this work, a potent and multifunctional platform has been developed by decorating 2D inorganic EB NSs on the surface of 3D‐printing CaP scaffolds to achieve both high‐effective OS elimination by photonic hyperthermia under NIR‐II (1064 nm) laser irradiation and the subsequently enhanced bone tissue regeneration. Particularly, EB NSs feature enhanced PTT effects activated by NIR‐II (1064 nm) laser irradiation which is superior to the extensively developed NIR‐I dependent PTAs. Especially, in addition to the destructive effects of PTT focused on the disease lesion, the mild temperature rise in the adjacent normal tissue can be comparatively curative, which promotes tissue regeneration such as osteogenesis and wound healing.^[^ ^42^, ^43^ ^]^ Meanwhile, Ca, Cu, and Si components from EB NSs and 3D‐printing CaP scaffolds would further synergize with each other to collectively enhance bone tissue regeneration. Importantly, mRNA transcriptome analysis was performed to identify the potential biological mechanisms. Results indicated that CaPCu‐scaffold‐based therapy could impact the gene expression pattern associated with cancer metabolism/progression and bone development. Taken together, the advanced CaPCu scaffolds exhibit promising clinical translation prospects in the elimination of OS by PTT and subsequently prompted osteogenesis as verified in vitro and in vivo.

Notably, the present work has the following merits. First, novel PTAs of 2D inorganic EB NSs have been synthesized and introduced into the treatment regimen of OS with enhanced PTT efficacy. Second, the successful integration of EB NSs and 3D‐printing bioactive CaP scaffolds further contribute to the development of advanced biomaterials coupling both cancer elimination and tissue rehabilitation. Third, since the application modality of as‐fabricated CaPCu scaffold is in situ implantation, which may also overcome limitations associated with the low tumor accumulation for the conventional PTAs. However, there is still room for the improvement of this work. For example, it would be better to explore more suitable animal models to verify the effectiveness of CaPCu scaffolds rather than evaluating the tumor elimination and bone regeneration on two separate models, since, in the clinic, they coexist in one disease (OS). To conclude, the as‐fabricated CaPCu scaffolds offer a preferable candidate for the treatment of OS and predict tempting prospects for further clinical translation.

## Conflict of Interest

The authors declare no conflict of interest.

## Supporting information

Supporting Information

## Data Availability

Research data are not shared.
