# Supplementary material for: Ultrathin 2D Inorganic Ancient Pigment Decorated 3D‐Printing Scaffold Enables Photonic Hyperthermia of Osteosarcoma in NIR‐II Biowindow and Concurrently Augments Bone Regeneration
Source: Adv Sci (Weinh). 2021 Aug 2;8(19):2101739. doi: 10.1002/advs.202101739 (PMC8498872; doi:10.1002/advs.202101739)
Supplement: Supplementary file 1 — Supporting Information [file ADVS-8-2101739-s001.pdf]

© 2021 Wiley-VCH GmbH

ADVANCED SCIENCE
Open Access

Supporting Information

for *Adv. Sci.,* DOI: 10.1002/advs.202001739

Ultrathin 2D Inorganic Ancient Pigment Decorated 3D-Printing Scaffold Enables Photonic Hyperthermia of Osteosarcoma in NIR-II Biowindow and Concurrently Augments Bone Regeneration

Chao He, Caihong Dong, Luodan Yu, Yu Chen* and Yongqiang Hao**

Ultrathin 2D Inorganic Ancient Pigment Decorated 3D-Printing Scaffold Enables Photonic Hyperthermia of Osteosarcoma in NIR-II Biowindow and Concurrently Augments Bone Regeneration

Chao He, Caihong Dong, Luodan Yu*, Yu Chen* and Yongqiang Hao*

C. He, Prof. Y. Hao
Shanghai Key Laboratory of Orthopedic Implants, Department of Orthopedic Surgery, Clinical and Translational Research Center for 3D Printing Technology, Shanghai Ninth People's Hospital, Shanghai Jiao Tong University School of Medicine, Shanghai, 200011, China.
E-mail: haoyq1664@sh9hospital.org.cn

Dr. C. Dong
Department of Ultrasound, Zhongshan Hospital, Fudan University, Shanghai 200032, China.

Dr. L. Yu, Prof. Y. Chen
Materdicine Lab, School of Life Sciences, Shanghai University, Shanghai 200444, China.
E-mail: yuluodan@shu.edu.cn; chenyuedu@shu.edu.cn

C. He and C. Dong contribute equally to this work.

A: Experimental section

Materials. $CuCO_3 \cdot Cu(OH)_2$ was obtained from Aladdin Chemistry Co., Ltd. SiO_2, $CaCO_3$ and Na_2CO_3 were acquired from Sinopharm Chemical Reagent Co., Ltd. Polycaprolactone (PCL) was purchased from Jinan Daigang Biomaterial Co., Ltd.

Synthesis and surface modification of CaPCu scaffolds. The EB ($CaCuSi_4O_{10}$) pigment was synthesized by a solid-state reaction method.[1] In brief, $CaCO_3$, SiO_2 and $CuCO_3 \cdot Cu(OH)_2$ in a molar ratio of 2:8:1 as starting materials and Na_2CO_3 (3 mol %) as a fluxing agent were hybridized in an agate mortar for 20 min. Then the mixture was calcined at 1000 °C for 24 h in air, and the products acquired were thoroughly ground into powder for further use. Then, the EB powder suspended in deionized (DI) water was treated by tip sonication for 96 h to obtain the EB NSs.

CaP scaffolds were fabricated utilizing 3D-printing. Briefly, 1 g PCL was dissolved in 10 g chloroform and then 6 g $CaCO_3$·powder was added into the solution to generate a homogenous cream for further 3D-printing (extrusion pressure: 0.45-0.55 MPa; inner diameter of the nozzle: 400 μm; rotation angle: 60°).

At last, the obtained CaP scaffolds were soaked in EB NSs absolute ethyl alcohol solution (25, 50 or 100 ppm) until the solvent volatilized completely at room temperature (RT). At this point, the final CaPCu scaffolds were constructed and ready for further evaluation.

Characterization. Transmission electron microscopy (TEM) photographs were acquired on a JEM-2100F electron microscope (200 kV). Scanning electron microscopy (SEM) images were taken by a field-emission Magellan 400 microscope. Hydrodynamic diameter distribution measurement was conducted on Zetasizer Nanoseries. The confocal laser scanning microscopy (CLSM) photographs were obtained on Zeiss LSM 710. The thermal imaging was recorded by an infrared thermal camera.

Cell culture and cell viability assay. The human OS cells 143B and HOS were obtained from American Type Culture Collection. The cells were cultured in Dulbecco's modified Eagle medium (DMEM) with antibiotics (100 $U \cdot mL^{-1}$ penicillin and 100 $\mu g \cdot mL^{-1}$ streptomycin) and 10% fetal bovine serum (Gibco, USA) in a humidified atmosphere with 5% CO_2 at 37°C.

Cells were firstly seeded on scaffolds (Φ 8 mm × 2 mm) in the 48-well plate with the indicated treatments. For cells under laser irradiation treatment, 1064 nm laser for 5 minutes was employed. After 24 h of incubation, the standard CCK-8 assay was conducted according to the instructions. For the live/dead cell staining, cells on the scaffolds were washed with PBS and then treated with PI solution (100 μL) and calcein-AM (100 μL) for 15 min before photographing. For FCM assay, the cells were detached by trypsin and stained by 7-AAD for 5 min before analysis.

In vitro osteogenesis differentiation. First, CaPCu scaffolds (Φ 8 mm × 2 mm) were placed into 48-well plates, and then 1 × 10^5 mBMSCs were seeded on them. The osteoinductive medium (OIM) contained β-sodium glycerophosphate (10^{-2} M), L-ascorbic acid (50 μg·mL^{-1}) and dexamethasone (10^{-7} M). After 7 d incubation, the adherent cells were detached by trypsin. The ALP activity assay and RT-qPCR were performed. The ALP activity was assessed using an ALP detection kit (Nanjing Jiancheng Bioengineer Institute, China) according to the instructions. The RNA of cells was extracted using TRIzol Reagent (Takara, Japan), and subsequently subjected to RT-qPCR with RT-qPCR kit (Takara, Japan) following the instructions. Relative quantification (RQ) was measured as $RQ=2^{-\Delta\Delta Ct}$. The sequences of the primers are summarized in **Table S1**.

Animal experiments. All animal experimental protocols were conducted following the relevant guidelines and approved by the ethics committee of Shanghai Ninth People's Hospital. Typically, Kunming mice (6–8 weeks), BALB/c nude mice (6–8 weeks) and Sprague–Dawley (SD) rats (8-10 weeks old) were purchased from Silaike (Shanghai, China).

For the *in vivo* biocompatibility evaluation, Kunming mice were randomly divided into three groups as the untreated healthy control mice (Ctrl), mice with CaP implantation (CaP) and mice with CaPCu implantation (CaPCu). After 30 days, mice were euthanatized and their blood and main organs were gathered for further evaluation.

For the *in vivo* PTT efficacy assessment, twenty BALB/c nude mice were involved in the establishment of OS tumor xenografts by injecting the 143B cells into the flank of mice. When the xenografts grew to 60 mm^3, mice were randomly separated into four groups as CaP, CaP + NIR, CaPCu and CaPCu + NIR. For NIR treatment, 1064 nm laser irradiation (1.0 W·cm^{-2}) for 10 min was executed 3 h post scaffold implantation. The tumor volume (V) was calculated by the formula: $V = 0.5 \times (\text{tumor length}) \times (\text{tumor width})^2$. The volume of each tumor and the bodyweight of mice were monitored weekly until the mice were euthanatized. Tumor tissue was then subjected to histopathological analysis.

For the bone regeneration observation, the calvaria defect model of rats was utilized. After the rats were anesthetized, a sagittal incision was made on the scalp. A 10 mm-diameter defect was established on craniums using an electric trephine drill. Then CaP or CaPCu scaffolds (n = 5) (Φ 10 mm × 2 mm) were implanted into the defect. Each rat was treated with antibiotics post-surgery. Moreover, the alizarin red and calcein were intraperitoneally injected at week 10 and 11, respectively. After 12 weeks, the rats were sacrificed and their calvarias were collected and analyzed by micro-computed tomography (μCT). After scanned by μCT, the calvarias were embedded in methyl methacrylate (MMA) for hard tissue slicing or decalcified for soft tissue slicing. The calcified samples were observed by CLSM and stained by Van Gieson's picrofuchsin staining, while decalcified samples were embedded in paraffin for H&E, Masson's and Goldner trichrome staining.

RNA-seq. RNA samples obtained from OS cells with CaP + NIR and CaPCu + NIR treatments were marked as Ctrl and Exp for short, respectively. Then, the samples were delivered to

Oebiotech Co., Ltd. for RNA-seq. Gene expression profiles were generated by the Htseq-count[2] and Cufflinks.[3] The differentially expressed gene lists output by DESeq were subsequently clustered according to GO and KEGG databases.

Statistical analysis. The mean, standard deviation (SD) and P values based on two-tailed t-tests were calculated with Excel (Microsoft). Differences were regarded significant at $P < 0.05$ (*$P < 0.05$, **$P < 0.01$).

B. Supplementary figures

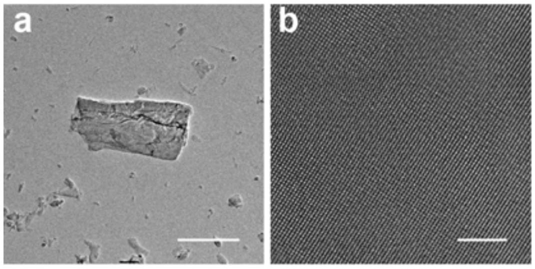

Figure S1. a) TEM image of ultrathin $CaCuSi_4O_{10}$ NSs. Scale bar, 1µm. b) High-resolution TEM (HRTEM) picture of ultrathin $CaCuSi_4O_{10}$ NSs. Scale bar, 5 nm.

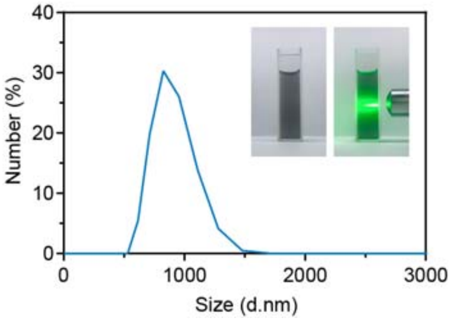

Figure S2. Hydrodynamic diameter distribution of ultrathin $CaCuSi_4O_{10}$ NSs analyzed by DLS. Inset shows the digital photos of ultrathin $CaCuSi_4O_{10}$ NSs dispersed in DI water and the related Tyndall effect.

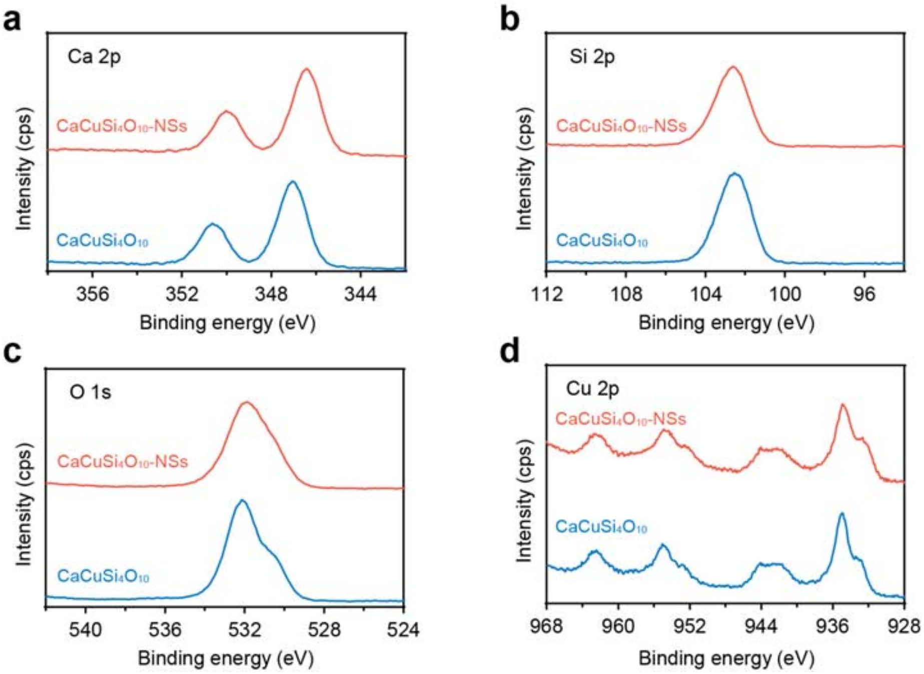

Figure S3. a) Ca 2p, b) Si 2p, c) O 1s and d) Cu 2p XPS spectra of $CaCuSi_4O_{10}$ and $CaCuSi_4O_{10}$ NSs.

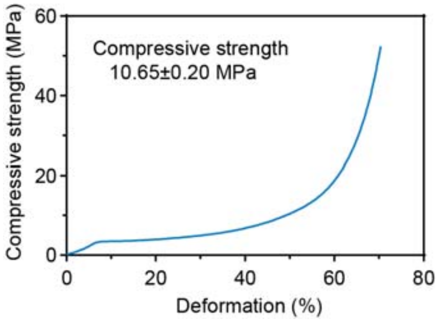

Figure S4. Compressive strength curve of the CaPCu scaffold.

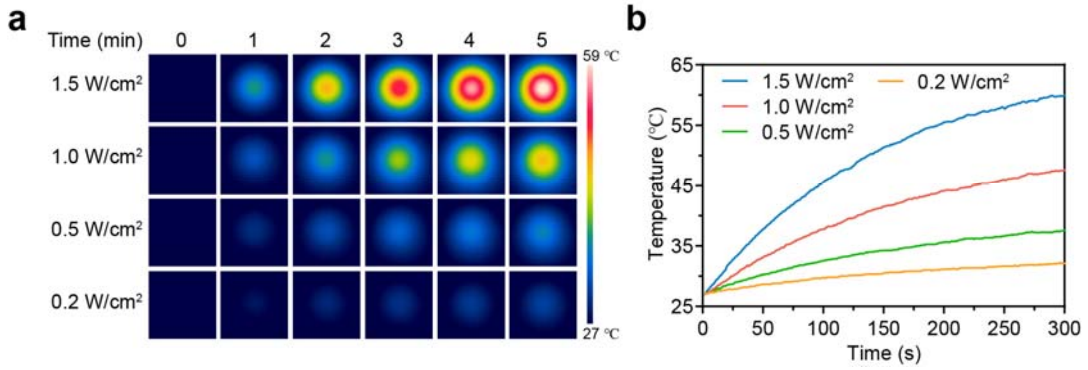

Figure S5. a, b) Photothermal images and curves of the CaPCu scaffold irradiated by 1064 nm laser at different laser power densities for varied periods.

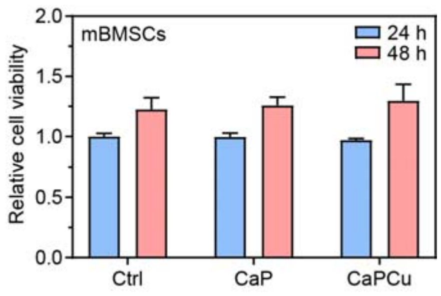

Figure S6. Relative viabilities of mBMSCs with the indicated treatments for 24 h or 48 h.

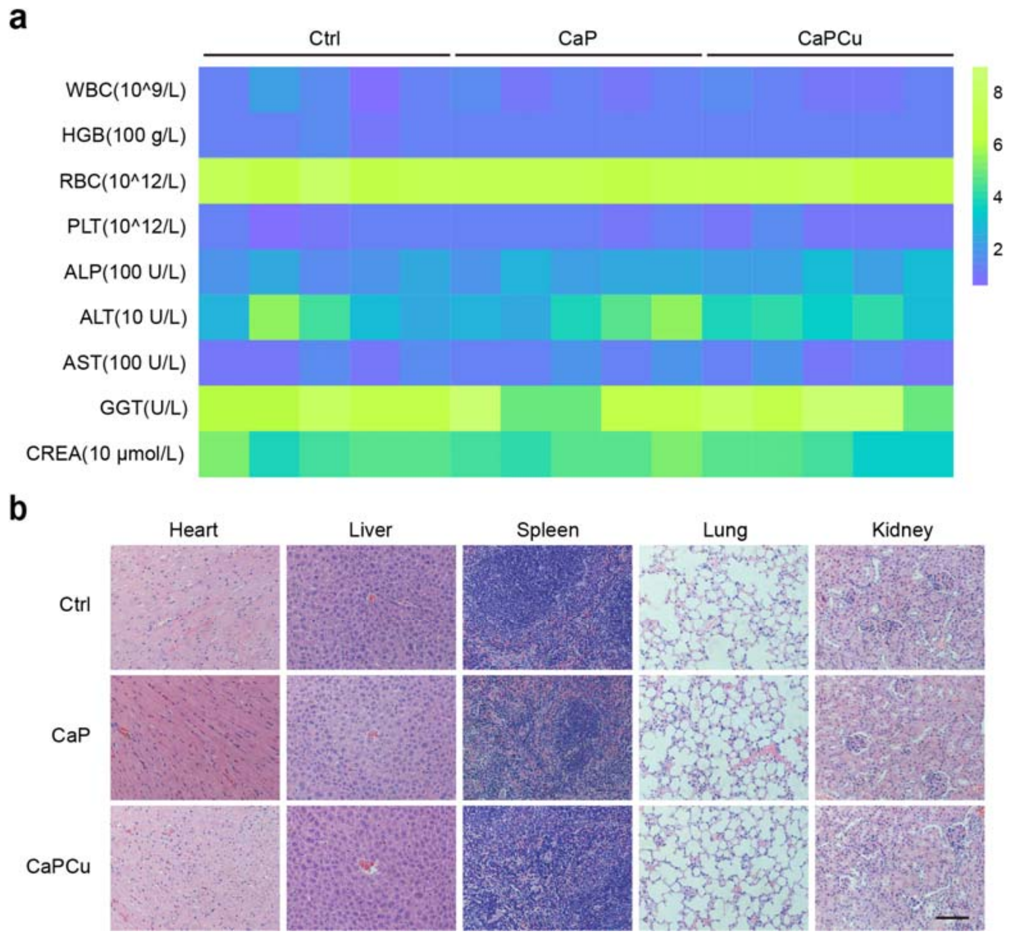

Figure S7. Toxicity evaluation of the CaPCu scaffold *in vivo*. a) Hematological and biochemical indexes of the mice with the indicated treatments after 30 days. b) H&E staining images of heart, liver, spleen, lung, and kidney obtained after mice were sacrificed after 30 days. Scale bar, 100 μm.

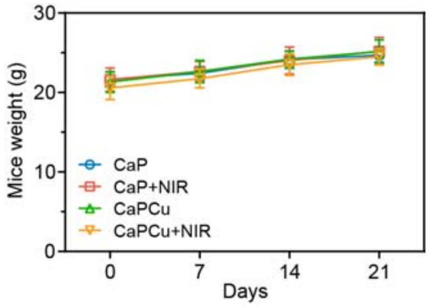

Figure S8. Time-dependent bodyweight of mice (n = 5) after different treatments.

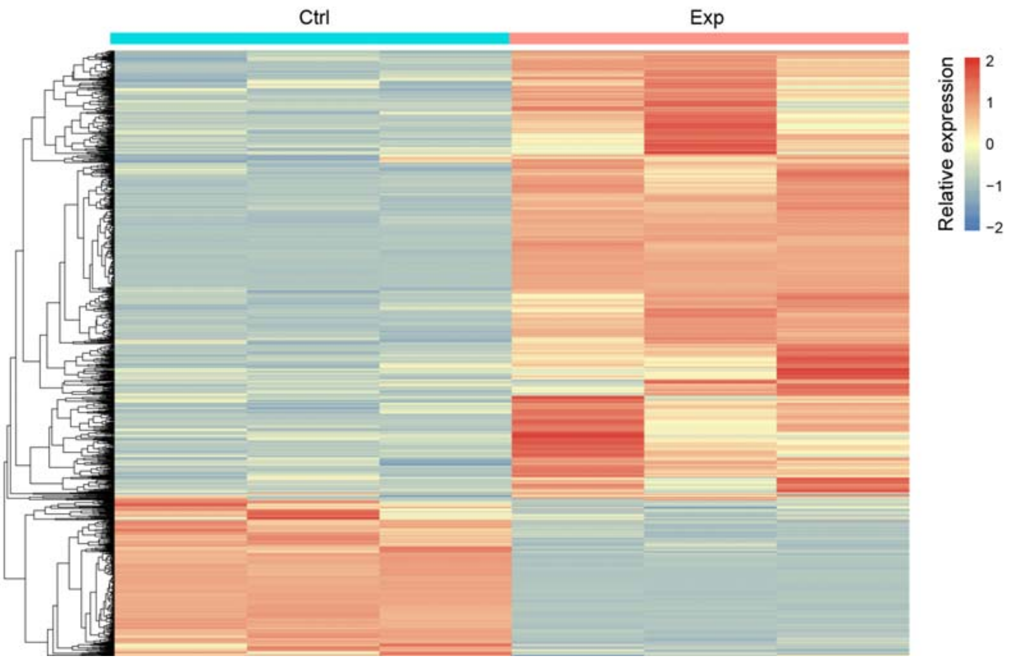

Figure S9. Heatmap of differential expression genes between the Exp and the Ctrl groups ($P < 0.05$, |fold change| ≥ 2).

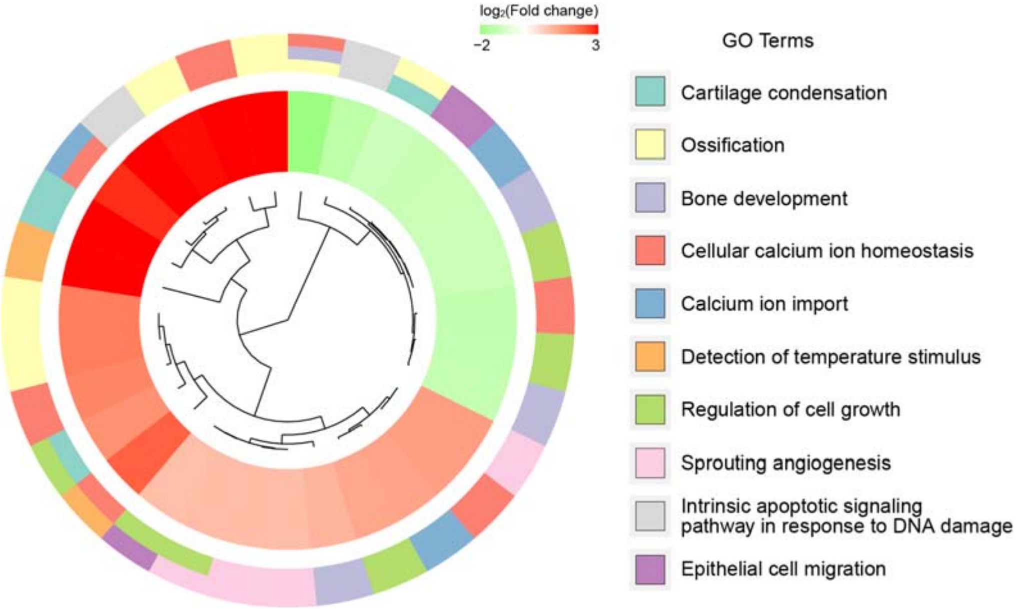

Figure S10. GO cluster analysis of differentially expressed genes between the Exp and the Ctrl groups.

C. Supplementary table

Table S1. Sequences of the RT-qPCR primers.

Gene name	Forward	Reverse
RUNX2	GACTGTGGTTACCGTCATGGC	ACTTGGTTTTTCATAACAGCGGA
OCN	CTGACCTCACAGATCCCAAGC	TGGTCTGATAGCTCGTCACAAG
BMP2	GGGACCCGCTGTCTTCTAGT	TCAACTCAAATTCGCTGAGGAC

Reference

[1] W. Chen, Y. Shi, Z. Chen, X. Sang, S. Zheng, X. Liu, J. Qiu, *J. Phys. Chem. C* **2015**, *119*, 20571.

[2] S. Anders, P. T. Pyl, W. Huber, *Bioinformatics* **2015**, *31*, 166.

[3] A. Roberts, C. Trapnell, J. Donaghey, J. L. Rinn, L. Pachter, *Genome Biol.* **2011**, *12*, R22.
